# Mapping populations at risk: improving spatial demographic data for infectious disease modeling and metric derivation

**DOI:** 10.1186/1478-7954-10-8

**Published:** 2012-05-16

**Authors:** Andrew J Tatem, Susana Adamo, Nita Bharti, Clara R Burgert, Marcia Castro, Audrey Dorelien, Gunter Fink, Catherine Linard, Mendelsohn John, Livia Montana, Mark R Montgomery, Andrew Nelson, Abdisalan M Noor, Deepa Pindolia, Greg Yetman, Deborah Balk

**Affiliations:** 1 Department of Geography, University of Florida, Gainesville, USA; 2 Emerging Pathogens Institute, University of Florida, Gainesville, USA; 3 Fogarty International Center, National Institutes of Health, Bethesda, USA; 4 Center for International Earth Science Information Network (CIESIN), Columbia University, New York, USA; 5 Ecology and Evolutionary Biology, Princeton University, Princeton, USA; 6 Demographic and Health Surveys, International Health and Development Division, ICF International, Washington DC, USA; 7 Department of Global Health and Population, Harvard School of Public Health, Boston, USA; 8 Office of Population Research and Woodrow Wilson School of Public and International Affairs, Princeton University, Princeton, USA; 9 Biological Control and Spatial Ecology, Université Libre de Bruxelles, Brussels, Belgium; 10 Fonds National de la Recherche Scientifique (F.R.S.-FNRS), Brussels, Belgium; 11 Research and Information Services of Namibia, Windhoek, Namibia; 12 The Population Council, New York, USA; 13 The International Rice Research Institute, Los Banos, Philippines; 14 Malaria Public Health and Epidemiology Group, Centre for Geographic Medicine, KEMRI - University of Oxford - Wellcome Trust Research Programme, Nairobi, Kenya; 15 School of Public Affairs, Baruch College, City University New York, New York, USA; 16 Department of Economics, Stony Brook University, New York, USA

**Keywords:** Population, Epidemiology, Demography, Disease mapping

## Abstract

The use of Global Positioning Systems (GPS) and Geographical Information Systems (GIS) in disease surveys and reporting is becoming increasingly routine, enabling a better understanding of spatial epidemiology and the improvement of surveillance and control strategies. In turn, the greater availability of spatially referenced epidemiological data is driving the rapid expansion of disease mapping and spatial modeling methods, which are becoming increasingly detailed and sophisticated, with rigorous handling of uncertainties. This expansion has, however, not been matched by advancements in the development of spatial datasets of human population distribution that accompany disease maps or spatial models.

Where risks are heterogeneous across population groups or space or dependent on transmission between individuals, spatial data on human population distributions and demographic structures are required to estimate infectious disease risks, burdens, and dynamics. The disease impact in terms of morbidity, mortality, and speed of spread varies substantially with demographic profiles, so that identifying the most exposed or affected populations becomes a key aspect of planning and targeting interventions. Subnational breakdowns of population counts by age and sex are routinely collected during national censuses and maintained in finer detail within microcensus data. Moreover, demographic and health surveys continue to collect representative and contemporary samples from clusters of communities in low-income countries where census data may be less detailed and not collected regularly. Together, these freely available datasets form a rich resource for quantifying and understanding the spatial variations in the sizes and distributions of those most at risk of disease in low income regions, yet at present, they remain unconnected data scattered across national statistical offices and websites.

In this paper we discuss the deficiencies of existing spatial population datasets and their limitations on epidemiological analyses. We review sources of detailed, contemporary, freely available and relevant spatial demographic data focusing on low income regions where such data are often sparse and highlight the value of incorporating these through a set of examples of their application in disease studies. Moreover, the importance of acknowledging, measuring, and accounting for uncertainty in spatial demographic datasets is outlined. Finally, a strategy for building an open-access database of spatial demographic data that is tailored to epidemiological applications is put forward.

## Introduction

The spatial modeling and mapping of diseases is increasingly being undertaken to derive health metrics, guide intervention strategies, and advance epidemiological understanding [1]. This has been driven by a recognition of the spatial, temporal, and demographic heterogeneities in disease risk (Table 1), and has resulted in significant recent methodological advances (for example [2,3]). Moreover, the need for estimates of populations at risk to guide funding allocations within the Millennium Development Goals (MDGs: http://www.un.org/millenniumgoals) framework continues to drive the development of disease mapping and modeling approaches.

**Table 1:** Heterogeneities in disease risks

**Heterogeneity type**	**Background information and examples**
Spatial	Understanding relevant spatial heterogeneities underlies our ability to map host risk of pathogen exposure. Predictions of disease importation or emergence are limited by our ability to distinguish disease-specific hotspots from continuous risk surfaces. Spatial variation in risk is defined by the specific biology of each host-pathogen relationship. Epidemiologically relevant spatial heterogeneities can be highly specific to each infection and must be correctly identified within the proper context of the ecology and landscape of each host-pathogen relationship. Spatial heterogeneities that impact risk profiles for exposure to a pathogen include large-scale environmental factors, such as temperature, access to water, and rainfall abundance, which can affect host susceptibility (e.g. within the African meningitis belt [4]), host exposure (e.g. proximity to malaria vector habitats [5]), and pathogen viability (e.g. cholera survival in the environment [6]). Within a population, the transmission events of infections drive the spatial progression of an outbreak after the initial exposure to the pathogen has already taken place. Transmission events are rarely observed and risk profiles must be constructed using proxies for transmission, again highlighting characteristics specific to each host-pathogen relationship. Risk profiles for directly transmitted diseases focus on host contacts between infectious and susceptible individuals. Important components of these contacts are host density, susceptibility, and mobility. Each of these factors can also be defined across spatial scales, from within household contact patterns to settlement-level risk factors. Urban and rural residence can be thought of as a basic (yet dichotomized) spatial heterogeneity that is closely associated with density and landscape, but typically urbanization has not been defined in spatial terms. Similarly, transmission of vector-mediated infections is impacted by spatial heterogeneities at the household and community level determined by host density, prevention measures, vector mobility and vector abundance. Spatial patterns of environmentally mediated infections will also be determined by the host-pathogen relationship.
Temporal	Epidemiologically important temporal heterogeneities will also be specific to each infection. For emerging infections, long-term changes in host settlements, habitat loss, and changing levels of interactions between humans and animal species interactions can define the risk of disease emergence over time [7] (e.g. ebola, SARS, monkeypox, HIV, H1N1 and H5N1 influenza). In other situations, seasonal and environmental factors may determine the population level risk of pathogen exposure (e.g. malaria vector habitats, hyperendemic areas of meningitis). Short-term risk of infection, or transmission of a pathogen within a population, is determined by the biology of the relationships between the host, pathogen and vector. These relationships establish the host susceptibility and infectious periods, and therefore the risk of transmission events. Population level susceptibility profiles (natural or derived) vary across temporal scales with respect to prior exposure and preventative measures. Temporal likelihood of transmission will be determined by length of exposure, and changes in abundance and susceptibility of the host and vector. Exposure and contact rates (density, migration) over the course of a day (as in commuter patterns for influenza [8]) are additional examples of temporal heterogeneities in transmission likelihood and risk across temporal scales.
Demographic and Socioeconomic	Susceptibility and transmissibility of infectious disease vary across differing demographic and socioeconomic groups due to differences in immunity, mobility, contact patterns and health status. Small-scale variations in socioeconomic and demographic factors can have a large influence on the geographical variation of infections compared to environmental factors. Age represents one of the most significant factors, with risk of morbidity and mortality of many diseases varying substantially across age groups. These include large variations in mortality and morbidity by age for malaria [9] and for clinical attack risk for dengue [10]. Heterogeneities in susceptibility and transmissibility also exist between the sexes, and especially during childbearing age for women, when pregnancy increases the risks of death for both the mother and fetus, and are important for diseases such as congenital rubella syndrome (CRS) [11]. At a population scale, differences in vital rates such as birth rates create heterogeneities in disease risk across space and time, as evidenced by rotavirus in the US [12]. For macro-parasite infections, such as helminths, in addition to environmental risk factors, the population at risk often depends on socioeconomic profiles and access to key infrastructure (housing quality, adequate sanitation and drinking water). For micro-parasite infections with human-to-human transmission, risk is again associated with individual socioeconomic attributes, but also with community/neighborhood attributes. In other words, the concentration of poverty or poor sanitation services increase risk, as evidenced by cholera outbreaks [13]. Finally, in addition to information on poverty status, knowledge of nutritional status is important; malnutrition can increase (i) susceptibility to many infectious diseases, (ii) the period of infectiousness (by reducing immune function and delaying recovery) and (iii) disease associated mortality [14].

Disease morbidity, mortality, and speed of spread vary substantially with demographic profiles, with clear risk groups and vulnerable populations existing. These have important implications for planning and targeting intervention strategies. The risk of pathogen infection to host populations exists at two spatial levels. First, there is a probability of initial exposure of a population to a pathogen, which defines the population risk. Second, there is a probability of transmission of a disease within a population, which defines the individual risk. Within these epidemic and endemic classifications, the implications for interventions vary across disease landscapes dependent upon the host-pathogen relationships.

Given the high degrees of individual and local heterogeneity within geographic regions or administrative units, effective policy design requires a detailed knowledge of the spatial distribution of relevant population attributes of interest, including size, age, gender, income, nutritional status, vaccination rates, or child mortality. From a public health perspective, detailed spatial datasets not only allow investigation of the relationship between policy inputs and individual-specific outcomes, but also build detailed and realistic predictive models and derive suites of health metrics. Disease mapping and spatial modeling studies have become increasingly detailed and sophisticated, with rigorous handling of uncertainties built in, but are limited when it comes to estimating populations at risk. Detailed spatial datasets on population distributions now exist, but maps of other demographic and socioeconomic characteristics to identify vulnerable subgroups remain lacking. To quantify these spatial variations in population attributes, recent high-impact studies have had to overlook subnational demographic variations in characteristics and rely on applying simple national-scale adjustments, e.g. [5,15-17].

The availability of high-resolution population data has increased dramatically through a series of global population mapping efforts over the past 15 years. Initially restricted to a few countries, location-specific population numbers have been made available for the globe over the past decades through the combined efforts of projects like the Gridded Population of the World (GPW) [18], the Global Rural Urban Mapping Project (GRUMP) [19], LandScan [20], and AfriPop [21]( http://www.afripop.org). All databases are in the public domain and allow individuals, companies, researchers, and policymakers to access population data either by administrative units or by user-specified geographic boundaries of interest. While the generation of these comprehensive population databases clearly constitutes a major achievement from a scientific perspective, two main factors limit the degree to which these databases can be used for research as well as for policy and planning: limited time frames and limited information on population attributes of interest. The first limitation is mostly the result of the irregular collection of detailed population data as well as the effort required in compiling global datasets at any given point in time. Given that most countries independently collect full censuses only once per decade and data sharing is complicated by a large set of copyright issues, most current population databases contain population data only on a five- or 10-year basis. When analyses warrant data for noncensus years, national growth rates [22], subnational growth rates from National Statistical Offices, or interpolation between available data points may be applied to produce estimates for intermediate years, as annual population fluctuations are generally limited.

The second constraint is more critical: little is known about characteristics of the underlying populations being mapped in detail. From a planning or research perspective, these factors can be of critical importance, as outlined in Table 1. Various freely available datasets exist to facilitate mapping improvements and add significant value to epidemiological analyses, but these remain scattered across different sources and require processing to be integrated into mapping. Here we review these sources of more detailed, contemporary, freely available, and relevant spatial demographic data, focusing on low-income regions of the world where disease burden is highest, and put forward a strategy for building an open-access database to link the various datasets, tailored to epidemiological applications.

### Usages of spatial demographic data in epidemiology

Population distribution datasets constitute an essential denominator required for many infectious disease studies. It is well known that disease transmission is spatially focal and heterogeneous (Table 1), partially due to the clustered nature of population distribution. The epidemiology of many diseases makes surveillance-based methods (reliant upon reporting from health facilities) for estimating populations at risk and disease burden problematic, particularly in low-income regions [23-25], while spatial heterogeneity in human population distribution can produce significant effects on transmission [3,26]. Cartographic and spatial modeling approaches have proven to be effective in tackling these factors (e.g., [27-29]). Such approaches can help characterize large-scale patterns of disease spread to evaluate intervention impact [3] and produce globally consistent measures of morbidity of known fidelity, which often represent the only plausible method in many African countries where surveillance data is incomplete, unreliable, and inconsistent [23,30,31]. As the precision and detail of disease risk mapping and modeling improves, spatial population datasets that capture these patterns are therefore required if populations at risk are to be more accurately quantified and disease spread among populations is realistically modeled for prediction and prevention purposes. Uses of gridded population count data in epidemiological studies are documented in Tatem et al. [1] and Linard and Tatem [32], and here we focus on studies that have attempted to incorporate spatial data on population subgroups.

Applications of gridded population datasets in epidemiology have involved estimating numbers of clinical cases, modeling the spatial progression of an epidemic, risk mapping and assessing the effects of urbanization, and the study of diseases ranging from dengue and yellow fever to HIV and leprosy. The majority of spatial modeling approaches of infectious diseases have been based on the environmental correlates of infection, due in part to the availability of high spatial resolution environmental data and relative paucity of spatial socioeconomic and demographic data. The most widespread uses of gridded population datasets in an epidemiological context have been in the study of malaria. Global spatial demographic datasets have been used to estimate populations at risk of malaria, which forms a fundamental metric for decision-makers at national and international levels [30,33]. While approaches for mapping malaria have become increasingly sophisticated (e.g., [2]), those for mapping population distributions have not kept pace, especially in low-income regions [1], where detailed spatial information on population composition is rarely available or utilized.

Previous studies that have aimed to enumerate vulnerable population subgroups at risk for different diseases have solely focused on utilizing simplistic national-level adjustments. The malaria burden in children under 5 years old was recently estimated based on a Zambia-wide survey and LandScan population data adjusted by a national-level estimate of the proportion of under-5 children [34]. Similarly, the numbers at risk of malaria globally in different age groups were estimated by applying national-level adjustments to GRUMP data [5,29,35,36]. Models of disease prevalence were overlaid onto population density maps adjusted by national-level proportions again to quantify school-age children and young adults at risk of schistosomiasis [17,37-40] and hookworm [38,41-43] and the number of pregnant women infected with hookworm in sub-Saharan Africa [44]. Specific estimates of populations at risk of malaria for pregnant women and children have also been derived from these maps, by combining GRUMP data with national-scale age, sex, and fertility data from the United Nations Population Division [15,45,46]. Finally, the numbers of children under 5 with anemia in West Africa were estimated using similar techniques [16]. In all of these examples outlined here, the problems of overlooking subnational variations in population through the national-level adjustments applied are illustrated in an example in the next section.

Spatio-temporal transmission models aim to simulate contacts between infectious and susceptible individuals and estimate the spatial spread of the disease. This helps to identify areas and times at risk of disease and assists in planning targeted interventions [47,48]. Sophisticated spatially explicit models have been developed to study the spatial progression of infectious diseases. Many of such spatially explicit models have made use of gridded population datasets as input data [3,49]. Gridded population data have also been used to develop agent-based simulation models at the regional level [28,50,51] and at the global level [52,53]. Whatever the spatial approach for modeling, population data are essential as these models, which generally require the generation of a virtual society with an appropriate distribution and composition of people [3]. Gridded data are preferred by these models in that the gridding process removes the irregularity associated with the native administrative units in which these data were initially reported and thereby makes the data more flexible for use with a variety of other spatial units or features. In addition, global (or continent-level) gridded population data provide valuable input datasets mainly because of their wide coverage, consistent spatial resolution, and availability in the public domain. Notably missing as discussed above is information on population attributes. This represents a limitation for models that can be substantially improved through the incorporation of realistic population attributes to build ‘synthetic’ populations. Previous studies have had to rely on national-level statistics or the application of census-derived attributes from one country applied to multiple others (e.g., [28]).

### Improving estimates of children under 5 years at risk of *Plasmodium falciparum* malaria

**Figure 1:**
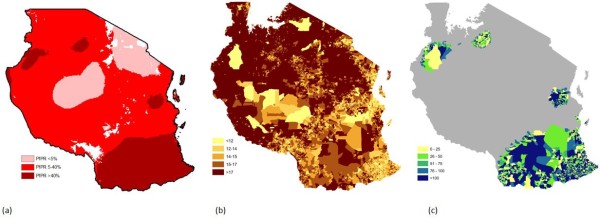
**For Tanzania in 2007**: (**a**) ***P. falciparum *malaria transmission classes (adapted from Hay**[5], measured by *P. falciparum* Parasite Rate (*Pf*PR), (**b**) percentage of residents under 5 years of age by ward, (**c**) percentage differences in estimates of number of children under 5 at risk of the highest transmission class by national- vs. ward-level adjustments.

The lack of availability of subnational spatial datasets on specific population groups that are particularly vulnerable to *P. falciparum* malaria has meant that simple national-level adjustments have been applied in influential studies to estimate the spatial distributions of, for example, children under 5 [15] or pregnant women [45,46] at risk. To illustrate the importance of mapping vulnerable populations to a level of spatial detail approaching that now used in disease mapping, here we compare estimates of under-5 children at risk of *P. falciparum* malaria in Tanzania in 2007 using transmission risk classes (Figure 1(a)) [5] overlaid onto a population distribution map ( http://www.afripop.org) adjusted to represent children under 5 by (i) applying a single nationwide percentage adjustment as defined by the UN’s World Population Prospects [22] (as undertaken in [15] – for Tanzania, percentage under 5 is estimated to be 17.9%) and (ii) applying per-district proportions of under-5 children derived from ward-level census data (Figure 1(b)).

Table 2 shows the difference in estimates of under-5 children residing in each transmission class, with large percentage differences found in each transmission class. We do not examine the spatial patterns of differences here, as this is beyond the scope of this analysis, but they remain an interesting area for exploration [1]. Overall, the adjustments from finer-scale age distributions indicate that national-level estimates substantially overestimate numbers at risk. Whereas Table 2 summarizes the overestimates by transmission classes, Figure 1(c) shows the spatial pattern of the misestimation in the highest transmission class. It shows the percentage differences obtained in estimates of under-5 children at risk of PfPR > 40% transmission level (mapped in Figure 1(a)) resulting from use of the ward-level map of children under 5 years rather than from applying a single nationwide adjustment. Most of these wards show differences above 25%, and several have discrepancies of greater than 100%. These malaria transmission maps and the populations at risk estimates derived from them are increasingly being used to guide planning, policy, and control. Such substantial differences in estimates of populations at risk, achievable through the use of an improved spatial demographic composition data, illustrate the urgent need to develop spatial databases of vulnerable populations.

**Table 2:** **Estimates of numbers of children under 5 at risk of *P. falciparum *malaria in Tanzania using the two differing demographic methods described in the text**

**Transmission Class**	**U5PAR1: UN Nationwide adjusted**	**U5PAR2: Census unit adjustments**	**Percentage change from U5PAR1 to U5PAR2**
*Pf*PR < 5%	770547	650174	−15.62175961
*Pf*PR = 5–40%	4315638	3383040	−21.6097365
*Pf*PR > 40%	773992	630518	−18.5368841

U5PAR = Under-5 population at risk.

### Spatial demographic data to meet needs

From an epidemiological and health metrics perspective, fundamental characteristics are age and sex. The most commonly needed age-sex specific groups in developing countries are: infants, children under 5, women of childbearing ages, and the elderly (Table 1). More specific needs might require the population of pregnant women, young adults, or urban children. Even though these numbers can generally be approximated by multiplying total population numbers by estimated national population fractions, the large epidemiologically important heterogeneity in population composition generated by migration and differential mortality and birth rates within countries and regions, and particularly between urban and rural residents, is likely to induce substantial degrees of imprecision in resultant output metrics (see previous section). The problem becomes even more severe when researchers or policy makers are primarily interested in nondemographic aspects of the population. In many cases, the main variable of interest may be a fraction of the population with certain health or behavioral characteristics: the number of children not vaccinated, the number of women without access to contraceptives, the number of children not going to school or not receiving formal health care. Many of these characteristics are not census-based, but rather can be ascertained through survey data, an aspect that we shall discuss in further detail below. Clearly it is not feasible for global population databases to generate on-demand maps for each of these factors on a regular basis, nevertheless the potential to leverage current freely available population databases appears large. Table 3 documents the principal datasets that are readily available without cost across multiple countries to achieve this.

**Table 3:** Sources of freely available spatial demographic data

**Data (standard survey name)/source**	**Time intervals**	**Typical spatial coverage**	**Typical strata**	**Relevant variables**
Census				
National Statistical Offices	Typically 10 years	Census enumerator area or courser level	Urban/rural, race or ethnic groups (often)	Sex, age, education, migration status, household and dwelling characteristics
Census Microdata				
https://international.ipums.org/international/	Typically 10 years	Admin 1-3	Urban/rural	Household and dwelling characteristics, sex, age, education, migration status, children ever born, children surviving
DHS (Demographic and Health Survey)				
Household, women 15–49, men 15–59, children born in the last five years				
http://www.measuredhs.com/	Varies by country, typically every 5 years	National, Admin 1/region, GPS coordinates of cluster locations for most recent surveys (last 15 years)	Urban/rural	Household and dwelling characteristics, sex, age, education, maternal and child health, fertility and full birth history, family planning, domestic violence, biomarkers, nutrition
MICS (Multi-indicator cluster survey)				
http://www.unicef.org/statistics/index_24302.html	UNICEF (Round 2, 1999–2001; round 3 2005–2007; round 4 is in the field 2009–present)	National, Admin 1	Urban/rural	Household and dwelling characteristics, sex, age, education, status, maternal and child health, child labor, domestic violence, summary birth history, anthropometry
LSMS (Living Standard Measure Survey)				
(Integrated Household Budget Survey and many others that are locally adapted)				
http://iresearch.worldbank.org/lsms/lsmssurveyFinder.htm	Irregular	National, Admin 1, some GPS coordinates	Urban/rural	Household and dwelling characteristics, sex, age, education, migration status,consumption, expenditures, income, nutrition,anthropometry, summary birth history
MIS (Malaria Indicator Survey)				
http://www.measuredhs.com/				
http://www.malariasurveys.org/	Varies by country, typically every 3 years	National, Admin 1/region, GPS coordinates of cluster locations for some surveys (last five years)	Urban/rural	Household and dwelling characteristics, sex, age, education, biomarkers
AIS (AIDS Indicator Survey)				
http://www.measuredhs.com/	Varies by country, typically every 3 years	National, Admin 1/region, GPS coordinates of cluster locations for some surveys (last eight years)	Urban/rural	Household and dwelling characteristics, sex, age, education, biomarkers
DHS (Demographic and Health Survey)				
Household, women 15–49, men 15–59, children born in the last five years				
http://www.measuredhs.com/	Varies by country, typically every 5 years	National, Admin 1/region, GPS coordinates of cluster locations for most recent surveys (last 15 years)	Urban/rural	Household and dwelling characteristics, sex, age, education, maternal and child health, fertility and full birth history, family planning, domestic violence, biomarkers, nutrition
MICS (Multi-indicator cluster survey)				
http://www.unicef.org/statistics/index_24302.html	UNICEF (Round 2, 1999–2001; round 3 2005–2007; round 4 is in the field 2009-present)	National, Admin 1	Urban/rural	Household and dwelling characteristics, sex, age, education, status, maternal and child health, child labor, domestic violence, summary birth history, anthropometry
LSMS (Living Standard Measure Survey)				
(Integrated Household Budget Survey and many others that are locally adapted)				
http://iresearch.worldbank.org/lsms/lsmssurveyFinder.htm	Irregular	National, Admin 1, some GPS coordinates	Urban/rural	Household and dwelling characteristics, sex, age, education, migration status, consumption, expenditures, income, nutrition, anthropometry, summary birth history
MIS (Malaria Indicator Survey)				
http://www.measuredhs.com/				
http://www.malariasurveys.org/	Varies by country, typically every 3 years	National, Admin 1/region, GPS coordinates of cluster locations for some surveys (last five years)	Urban/rural	Household and dwelling characteristics, sex, age, education, biomarkers
AIS (AIDS Indicator Survey)				
http://www.measuredhs.com/	Varies by country, typically every 3 years	National, Admin 1/region, GPS coordinates of cluster locations for some surveys (last eight years)	Urban/rural	Household and dwelling characteristics, sex, age, education, biomarkers

**Figure 2:**
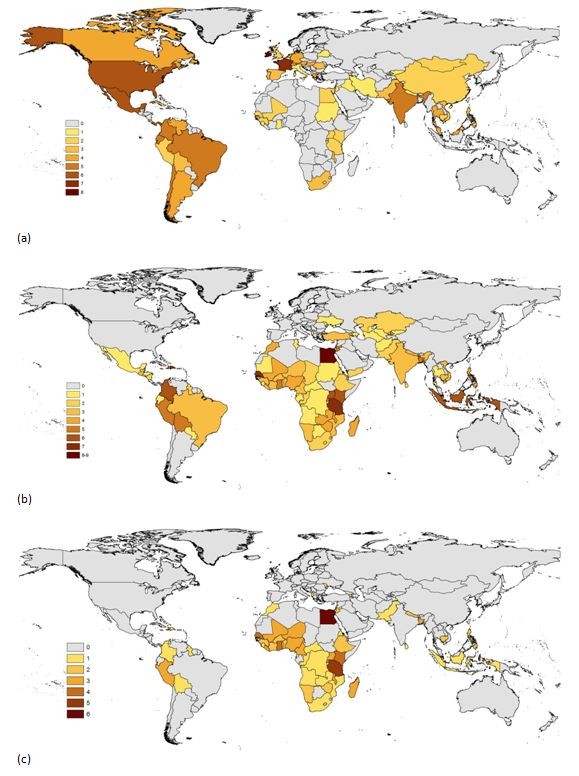
**Maps showing the availability of useful demographic datasets for deriving subnational estimates of population attributes.** (**a**) Numbers of census microdata records maintained at the International Public Use Microdata Series repository ( https://international.ipums.org/international/), (**b**) combined numbers of Demographic and Health Surveys (DHS), Malaria Indicator Surveys (MIS), and AIDS Indicator Surveys (AIS) conducted for each country, (**c**) combined numbers of DHS, MIS, and AIS with GPS cluster coordinates available.

Census data form the basis of existing spatial demographic databases [19,20], and such population and housing censuses are undertaken for almost all countries in the world, including developing countries, generally every 10 years (the date of past and upcoming planned censuses are available here: http://unstats.un.org/unsd/demographic/sources/census/censusdates.htm), but these provide only population counts. A range of other population-attribute information is generally collected during population censuses such as age, gender, urban/rural residence, and migration information, and, for the majority of countries, made available in some form on national statistical office websites. This information supplies a series of single population characteristics at whatever level of geographic detail is made available by the National Statistical Office. Often, this information is available through data tables aggregated at coarse administrative levels, however, and full-detail datasets can be difficult to obtain. An addition to the aggregated full census data are large samples of household-level records derived from censuses (census microdata) that provide age and sex structure, as well as many other compositional measures, reported generally by administrative level 1 (e.g., province) or 2 (e.g., district). These data keep information about households intact so that combinations of variables can be made. The largest repository of such data is the International Public Use Microdata Series ( https://international.ipums.org/international/) and the data held there are mapped in Figure 2(a).

While census aggregates and census microdata samples are typically large enough to cover small or moderately sized geographic areas, they are only carried out approximately every 10 years and are limited in content. Survey data offer much richer content on shorter time intervals but are limited in spatial coverage (e.g., Figure 2(b)). In most high- and low-income countries, geo-referenced household-based surveys are collected on a regular basis. These surveys contain detailed local population characteristics for a finite number of locations, which could be used to generate characteristic-prevalence surfaces for a given country and year. Overlaying these characteristic surfaces with population estimates would likely become an invaluable tool both for researchers and policymakers.

Data on a rich variety of population attributes can be obtained from a range of international household survey programs, each of which is listed in Table 3. These provide subnational urban (or rural) age and sex structures, educational compositions, employment information, and countless other socioeconomic and health indicators at the level of subnational regions. Large household survey programs such as the Multiple Indicator Cluster Surveys (MICS) and Demographic and Health Surveys (DHS) listed in Table 3 are reasonably well standardized and cover many low-income countries (Figure 2(b), with multiple rounds in each country. Additionally, in most recent DHS data sets, the survey clusters have been geo-coded (Figure 2(c)). In order to protect the confidentiality of survey respondents, cluster locations are randomly displaced by up to 2 km in urban areas and 5 km in rural areas. Moreover, in rural areas, the DHS cluster locations provided can represent large and potentially heterogeneous areas. The existing approaches using DHS data have not taken advantage of spatial modeling to expand the use of DHS data below the survey region level (usually administrative level 1). The geocoded cluster data from the DHS allow for data regrouping to different levels of representativeness while still respecting the sample frame. While MICS now regularly collect geocoded data, these datasets are not available to enable mapping of the data at finer level than the survey region level.

Each of the datasets described here and listed in Table 3 report demographic information aggregated by named administrative units. Rarely, however, are spatial data on the boundaries of these units provided with the data. For spatial analysis, therefore, GIS boundary datasets must be found that match the reported administrative units. This is often a nontrivial task given regular boundary changes over time, alternative names, and mismatches with national boundaries. The initiation of open access repositories of standardized administrative boundary datasets (e.g., GADM: http://www.gadm.org/), and documented histories of changes (e.g., http://www.statoids.com/) simplifies such operations. Moreover, DHS also shares the geography for their surveys on request.

### Designing a spatial demographic database

**Figure 3:**
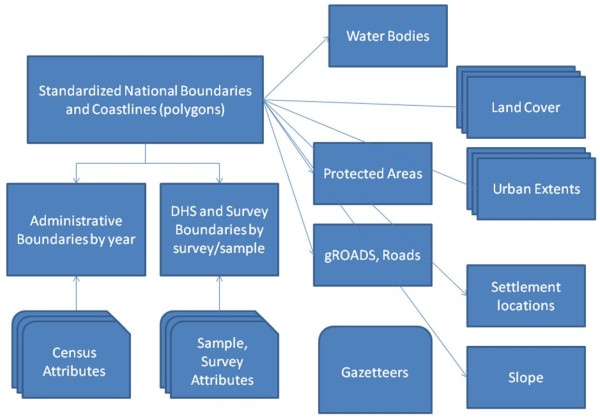
**Design of a relational spatial demographic database.** Table 4 provides details on each layer.

The datasets described in the previous section are presently scattered across disparate sources (Table 3). To better fulfill the needs of disease modeling and cartographic-style derivations of health metrics, we propose the construction of a spatial database. The construction of this database would involve not only the housing of the disparate demographic datasets in a central open access location, but also their linkage to GIS datasets to enable the construction of spatial datasets representing a variety of epidemiologically relevant variables. The recent development of spatially enabling tools for database servers, such as PostGIS ( http://postgis.refractions.net), which provides support for geographic objects in object-relational databases, provides the ideal framework for construction of the database. The database would be hosted on a central server and accessed through an interactive web portal. Table 4 outlines the spatial datasets that would be included in a database to spatially reference the datasets in Table 3 and provide additional information to increase mapping capabilities. The framework spatial data are open-access GIS datasets that can be reused by multiple organizations for different purposes. Figure 3 outlines how these datasets link together in the relational spatial database. The key objectives of this database would be to:

1. Provide disaggregated spatially-referenced data on population sizes and characteristics such as age, sex, urban/rural location, and education

2. Facilitate data sharing between differing platforms and demographic mapping projects

3. Provide a high degree of transparency, documentation, and flexibility with respect to data sources and the treatment of uncertainty

**Table 4:** Components of relational spatial demographic database based on freely available datasets

**Feature**	**Example dataset**	**Example dataset source**
National boundaries	SALB	http://www.unsalb.org
Administrative boundaries	GADM	http://www.gadm.org
DHS boundaries	MEASURE DHS	http://www.measuredhs.com
Coastlines	GBWD	http://dds.cr.usgs.gov/srtm/
Water bodies	SWDB	http://dds.cr.usgs.gov/srtm/version2_1/SWBD/
Land cover	GlobCover	http://www.ionia1.esrin.esa.int
Protected areas	WDBPA	http://www.wdpa.org
Urban extents	MODIS	http://www.sage.wisc.edu/people/schneider/research/data.html
Settlement locations	NGA Geonames	http://www.earth-info.nga.mil/gns/html
Elevation and slope	SRTM	http://www.srtm.csi.cgiar.org
Infrastructure	gRoads	http://www.ciesin.columbia.edu/confluence/display/roads/Global±Roads±Data

The database is designed to encourage data sharing, built in a manner that can be replicated across different nodes, with standardized, agreed-upon representations. For example, whilst the GRUMP and AfriPop project outputs take different forms and use differing modeling techniques, each is built upon standard representations (national boundaries, coastlines, administrative units) and aim to use the most detailed and contemporary population data available. A standardized database framework would encourage sharing of new and improved datasets between projects, benefitting a range of user groups. By building in differing levels of access control, new datasets can be reviewed and processed before release to a wider user community, and also datasets that remain copyrighted can be controlled in their accessibility.

Documentation of all aspects of the data and database structure are key to ensuring ease of use, integration with epidemiological applications, and accessibility to a wide user community. This will focus around database version control, the development of a data dictionary, with full documentation of the datasets archived within it, and metadata accompanying the GIS-related datasets. The distinction between spatial metadata and a data dictionary must be made: they are much different and both are necessary. A data dictionary is needed to understand the shortened name and values of particular variables, for example, whereas the metadata speak to the spatial lineage and quality of the data. Some institutional or database history is sometimes warranted, for example, when data collection for a given variable has changed. Ensuring that this documentation is oriented toward the user through full explanation of assumptions made and quality issues with the data provided will be important. Moreover, the construction of a library of tools and techniques for analyzing and using the data within the database using a forum and other mechanisms, such as a code repository, will facilitate ease of use. Finally, the provision of quantitative measures of data quality and uncertainty will be of great importance. This could range from basic information of the timeliness, resolution, spatial uncertainty, and standard errors of datasets that enable informed interpretation of resulting mapped products to the more rigorous handling and measurement of uncertainties (see next section).

### New methods, data, and future challenges

We have so far outlined datasets and a basic framework for compiling them to meet immediate disease modeling and health metric demographic needs. Several opportunities and possibilities exist however with which to supplement and improve the scope and accuracy of resources available. Here we outline these briefly, along with future challenges likely to be faced in implementation.

#### Urban populations

The health divide between urban and rural populations has been well documented [54], as have the increasing levels of urbanization around the world [55]. While much has been done in the past 10 years to refine population data that delineates urban areas [19,56,57], much less is known about populations within cities even within relatively coarse divisions (city center, suburban, peri-urban) or by slum dwellers and others (much also remains to be learned about population distribution within rural areas). Africa, in particular, will undergo rapid urbanization in the coming decades [55], yet the data record to understand the demographic variation and health conditions in these cities, let alone changes in disease transmission that may result from urban change, is largely absent. If it is important to understand what is happening within urban areas, even the currently available cluster-level data of the DHS program (Figure 2(c)) is inadequate. While the DHS program and some other surveys have focused on collecting larger urban samples, there remains a need for large samples of urban populations to permit city-specific analyses. Additionally, the definition of urban is not standard across the DHS countries. There is reason to believe that even greater heterogeneity of health and socioeconomic characteristics exist within urban areas.

As with the demographic datasets discussed already, there are a variety of disparate spatial datasets on urban populations that could be brought together to get a better perspective. City population counts for cities with populations of 100,000 and above are produced by the UN Population Division World Urbanization Prospects [55]. Alternative sources of city and settlement population sizes include the City Population website ( http://www.citypopulation.de), while different projects are focused on mapping city extents, which these counts could be matched to (e.g., [19,58], http://www.afripop.org). There still exist significant gaps, however, such as time-series of urban spatial extents, which would facilitate the development of ways to forecast changes in urban extents. Also, information on properly defined neighborhoods within cities is important, such as within-household and within-neighborhood population density, but so are other contexts (e.g., schools).

#### Subnational spatial and temporal projections

Most low-income countries do not produce population projections, or forecasts, at a subnational level. Even the United Nations Population Division’s urban population projections [55] do not produce city-level population projections. Yet the demographic inputs for generating subnational estimates and projections are increasingly becoming available. Subnational projections are now being undertaken at least for very large countries (for example, India and China) and for small and large cities in the developing world [59]. For the latter, the forecasting method departs from the traditional cohort-component method and instead uses longitudinal data on cities and subnational estimates of demographic rates (urban fertility, mortality, and migration) derived from survey and census microdata in an econometric model of city growth [60]. These new approaches depend on harnessing old data in a spatial framework. The spatial framework allows disparate units to be linked in new ways, yielding new estimates and projections. Because these methods are largely probabilistic and derived from modeling exercises, the uncertainty associated with these estimates should also be characterized.

#### Quantifying uncertainty

The variety of ages, spatial resolutions, and sample sizes of input demographic data translates to great variations in accuracies and uncertainties of any output gridded demographic data products, and this is rarely acknowledged [1]. The most basic level of quantification and communication of this uncertainty to users involves the provision of information on input datasets and methods used in construction, such as is undertaken for GPW, GRUMP [19], and AfriPop [21] ( http://www.afripop.org). Ideally, a more rigorous quantification of the uncertainty inherent in output gridded demographic datasets should be undertaken. The rigorous handling and propagation of uncertainty through a mapping process is now regularly undertaken in disease risk mapping within a Bayesian framework (e.g., [2,16,17]), resulting in full posterior prediction distributions for each grid cell, providing flexibility in the derivation of differing uncertainty metrics and enabling the production of accompanying uncertainty maps. Undertaking an equivalent approach for deriving accompanying uncertainty maps for demographic datasets would require consideration of the input datasets and output requirements. For instance, this could take the form of estimating the uncertainty in gridded population distribution mapping from census data summarized by administrative unit. Here, two of the major sources of uncertainty are the age of the census data in relation to the output prediction year and the size of the administrative units relative to the population sizes within them and output grid cell size. While uncertainty in temporal projection of census data is relatively well studied, the spatial aspect remains unexplored. For example, gridding a 50,000 km^2^ administrative unit containing 1 million children under age 5 to 30 arc second resolution results in greater uncertainty about the population size and composition residing in each grid square than does the same resolution gridding of a 1,000 km^2^ administrative unit containing 10,000 children under age 5. Based on simulating all possible permutations of grid square composition, bound by the limits imposed by the original administrative unit size and vulnerable population totals, per-grid square measures of spatial uncertainty in composition that relate directly to the gridding methodology could be derived. Secondly, the availability of the DHS cluster locations opens up the possibility of estimating surfaces of variables with associated uncertainty, and this is discussed below.

#### Gridding household survey data

The availability of the GPS coordinates of DHS clusters (Figure 2(c)) has prompted several studies to utilize geostatistical approaches to derive continuous estimated surfaces of variables of interest. However, survey data are generally collected to be nationally representative and, as such, their sampling frames may not lend themselves to finely resolved geographic grids. The DHS program has been a leader in collecting and providing geocoded information of the survey clusters in addition to their standard data files in which data can be tabulated by first-order subnational regions as well as urban/rural classification. Early examples have demonstrated the value of such approaches for deriving continuous maps of variables of interest from geolocated DHS cluster data. For example, Gemperli et al. [61] investigated spatial patterns of malaria endemicity as well as socio-economic risk factors on infant mortality in Mali using a Bayesian hierarchical geostatistical model. Meanwhile, Soares and Clements [16] used a similar approach for anemia mapping. However, these approaches did not take account of the DHS sampling design or the random spatial displacement that cluster data undergo, and overcoming these issues should be a priority for future applications [62]. Apart from utilizing the cluster location, the subnational regions supply information that can be used with more finely resolved grids. One approach that uses spatial coverage of census aggregates combined with the attribute breadth in survey data is that of the Poverty Mapping efforts [63]. But this is not necessarily the only approach to consider.

#### Migration and mobility mapping

Very little is known about migration and mobility within countries, which may occur seasonally and periodically as well as permanently, except through case studies and qualitative place-specific analyses. Disease modeling and health metric derivation, as well as demographic analyses, increasingly require information on migration and mobility [64-66]. These data are the weak link of the demographic record – even the stock estimates of subnational migration have been largely ignored. Disease modelers often want to know about daily movements rather than decadal ones, but the decadal moves may be important to evaluate for changes to place-specific vulnerability of residents. Decadal moves should be examined more closely with existing survey and census microdata; characterizing more frequent moves will require data collection methods that depart from the standard demographic tool kit. Use of new data, such as spatial locations derived from GPS tracking devices [67] and cell phone usage [68] may show promise. However, to be useful, methods for using these data in combination with more standard demographic data will be necessary.

## Conclusions

Growing trends in research and funding for disease mapping and spatial modeling to derive health metrics and guide strategies are increasing needs for spatial demographic data of similar scope and quality for use in estimating sizes and characteristics of populations at risk. However, existing spatial demographic databases are often based on coarse resolution and outdated input and lack any consideration of population attribute mapping. These drawbacks are likely contributing to substantial uncertainties in disease modeling and health metric outputs [1]. Here we have shown that datasets to rectify this exist but remain scattered across multiple repositories and websites, requiring collation into a central open-access database to become more widely used and build on the strengths of each data type, overcoming temporal, spatial, and attribute limitations. We have put forward a basic database design here to achieve this and lay the foundations for undertaking detailed mapping of population attributes for providing spatial demographic data in disease studies.

## Competing interests

The authors declare that they have no competing interests.

## Author's contributions

AJT conceived and designed the manuscript. All authors contributed to writing the manuscript and have read and approved the final manuscript.
